# ZC3HAV1 promotes the proliferation and metastasis via regulating KRAS in pancreatic cancer

**DOI:** 10.18632/aging.203296

**Published:** 2021-07-28

**Authors:** Wei Huang, Hao Hua, Guoliang Xiao, Xianjin Yang, Qin Yang, Lu Jin

**Affiliations:** 1 Department of Gastrointestinal Surgery, The First People’s Hospital of Neijiang, Neijiang, Sichuan, China; 2 Department of Hepatic-Biliary-Pancreatic Surgery, The First People’s Hospital of Neijiang, Neijiang, Sichuan, China; 3 Department of Gastroenterology, The First People’s Hospital of Neijiang, Neijiang, Sichuan, China; 4 Department of Pediatrics, The First People’s Hospital of Neijiang, Neijiang, Sichuan, China

**Keywords:** pancreatic cancer, ZC3HAV1, proliferation, metastasis, KRAS

## Abstract

Proliferation and metastasis are important malignant features of pancreatic cancer (PC), but the underlying molecular mechanism is unclear. ZC3HAV1, a PARP family member of proteins-enzymes, has been considered to play a significant part in a variety of biological processes. Nonetheless, the functions of ZC3HAV1 in developing PC are still unknown. This research aims to explore the biological function and the expression of ZC3HAV1 shown in PC. In our study, PCR analysis suggested that ZC3HAV1 was expressed at a high level in PC tissues and cell lines, and high ZC3HAV1 expression was remarkably related to poor prognosis. The functional assays indicated that upregulated ZC3HAV1 accelerated PC cell proliferation along with colony formation capacities *in vitro*. Subsequently, ZC3HAV1 could upregulate cyclin D1 and CDK2 and also promote G1/S transition in cells of PC. What’s more, we also discovered that ZC3HAV1 promotes the migration and the invasion of PC cells. It upregulates the expression of EMT (epithelial-mesenchymal transition) relevant markers. Conversely, the functional assays showed that ZC3HAV1 knockdown significantly reduced tumorigenesis. Using bioinformatics analysis and immunoprecipitation assays we found that ZC3HAV1 could directly bind to KRAS and positively regulate its expression. Furthermore, ZC3HAV1 overexpression activated MAPK signaling by increasing p-ERK levels. Conversely, knockdown of KRAS attenuated ZC3HAV1-mediated promotion of proliferation and invasion in cells of PC. The result indicated that ZC3HAV1 was in relation to poor prognosis and accelerated the proliferation and metastasis of PC cells by regulation of KRAS. Our research may offer brand-new evidence to diagnose and treat PC in clinic.

## INTRODUCTION

The definition of pancreatic cancer (PC) is a type of digestive system tumor with high level of malignancy. Since people’s living standard has increased while their diet and living habits become worse, the morbidity of PC has grown by six times in the last two decades [1, 2]. Surgical resection remains to be the first choice for treating PC. Even if patients have already taken surgery, their survival rate for five years is merely 8.77% and meanwhile the recurrence rate remains 54% within 1 year. The foremost reason lies in the high degree of malignancy, and strong invasion, and it might be related to lymph node metastasis together with nerve vascular infiltration at an early phase. As a result, multiple patients are diagnosed with an end-stage disease, and local and distant metastasis can occur quickly [3, 4]. The situation of PC is getting worse. Therefore, better knowledge of the underlying molecular mechanism of PC will facilitate to identify new diagnostic, prognostic markers and development of new therapeutic strategies.

ZC3HAV1, a zinc-finger antiviral protein, goes by the name of ARTD13 or PARP13 and belongs to PARP protein family. Recently, according to numerous reports, the PARP protein families get involved in developing all kinds of diseases, such as cancers [5, 6]. For example, according to the report, PARP10 accelerates cell proliferation together with tumorigenesis through mitigating replication pressure. As another example, PARP3 is a brand-new therapeutic goal which can change Rictor/mTORC2 signaling as well as promote progression of tumor in BRCA1-related cancers. Moreover, PARP9 is overexpressed in mankind breast cancer. It accelerates cancer cell metastasis [7–9]. First spotted in a screening for antiviral factors, ZC3HAV1 is composed of four N-terminal RNA-binding CCCH-type zinc finger domains which exist in proteins involved with regulating of RNA stability. ZC3HAV1 is also addressed as zinc finger antiviral protein, playing vital parts in immunity and microRNA mediated stress responses [10, 11]. In addition, it’s reported in recent studies that ZC3HAV1 is in relation to the development of colon cancer, liver, cancer and bladder cancer, which suggests that ZC3HAV1 may be beneficial to the progression of some cancers [12]. However, ZC3HAV1 expression and function in PC has rarely been reported.

KRAS belongs to the RAS gene family, encoding the K-Ras protein. Studies have shown that 30% of human tumor is related to the mutation of RAS gene. The products after mutation can be activated continuously, and participate in the survival, proliferation, migration, proliferation as well as angiogenesis of cancer cells. Many researches indicate that KRAS plays a role in behaving as a target gene in tumors [13, 14]. For example, overexpressed let-7a suppresses glioma cell malignancy by taking aim at KRAS directly, which is independent of PTEN [15]. Besides, downregulation of miRNA-126 conduces to tumorigenesis of squamous tongue cell carcinoma by aiming at KRAS [16].

This study is designed with the purpose of finding out potential function of ZC3HAV1 in mankind PC cells. In such a study, firstly we revealed that ZC3HAV1 was upregulated, and the overexpression of ZC3HAV1 promoted the proliferation along with the metastasis of PC cells via binding and upregulating the expression of KRAS and activating ERK signaling. In addition, downregulation of KRAS expression could reverse the promotion influence of ZC3HAV1 on the proliferation and metastasis of PC cell. Mechanically, ZC3HAV1/KRAS/ERK signaling axis might regulate the proliferation and migration of PC cell. Our study offered a novel perspective for the diagnosis as well as the cure of PC.

## RESULTS

### Expression level of ZC3HAV1 was up-regulated in PC and in relation to poor prognosis

**Figure 1:**
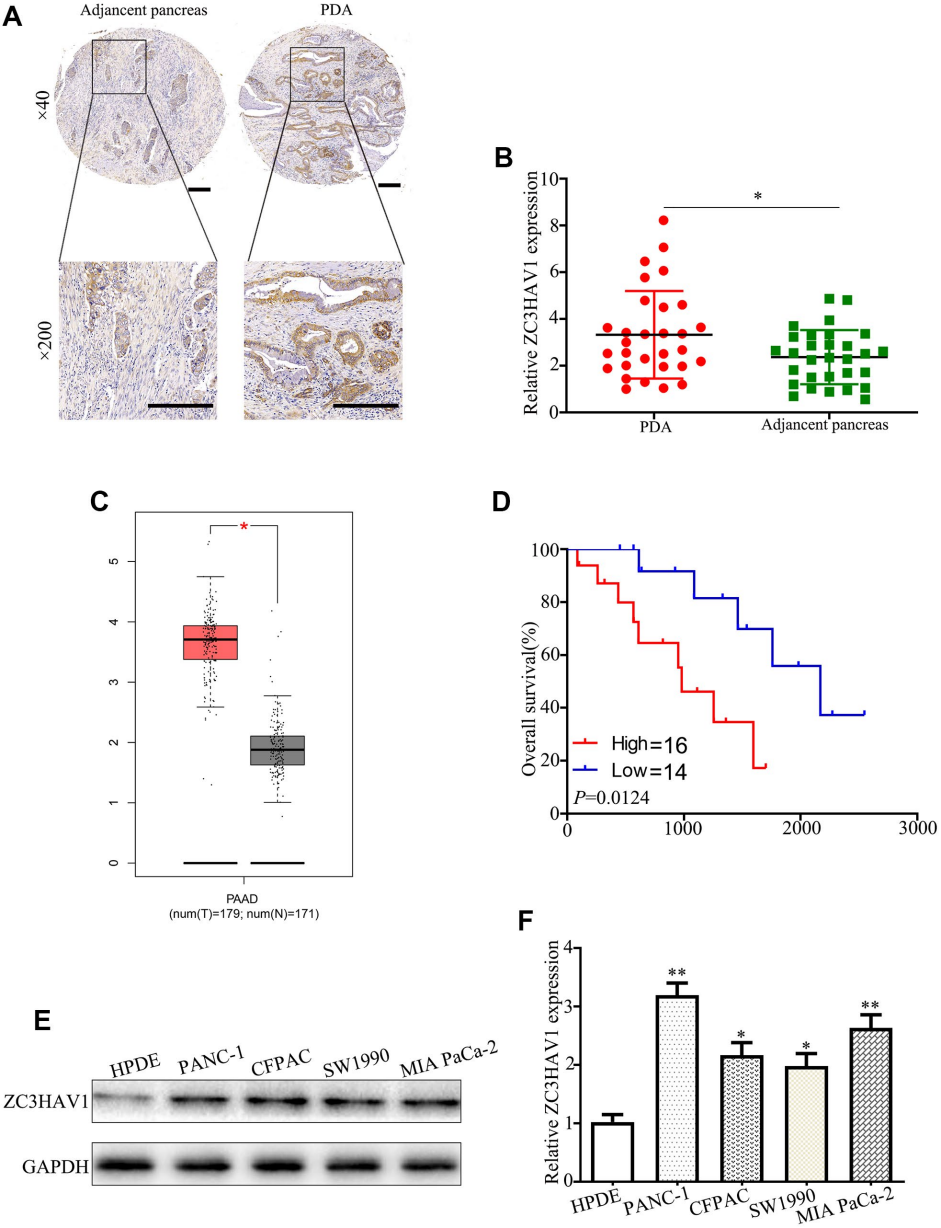
**The associated expression of ZC3HAV1 was up-regulated in mankind PC tissues and cells and demonstrated poor prognosis.** (**A**) Representative IHC images of ZC3HAV1 expression in PC tissues and matched adjacent non-tumor tissues (n=30) (scar bar: 50 μm). (**B**) Expression of ZC3HAV1 mRNA in 30 paired PC tissues and adjacent normal tissues was determined by qRT-PCR. (**C**) The expression of ZC3HAV1 in PC tissues and adjacent normal tissues built on the GEPIA database. (**D**) Kaplan-Meier survival curve generated in accordance to the ZC3HAV1 expression level. (**E**, **F**) The mRNA and protein expression of ZC3HAV1 in PC cell lines and in human pancreas ductal epithelial cell line (HPDE). Data appeared to be average ± SD, *P< 0.05, **P< 0.01.

In order to explore potential significance of ZC3HAV1 in PC, firstly we examined its expression pattern in PC tissues via qRT-PCR along with IHC staining. The level of ZC3HAV1 was remarkably up-regulated in PC tissues in comparison to the adjacently normal tissues (Figure 1A, 1B). In addition, an analysis was performed by us in the Gene Expression Profiling Interactive Analysis (GEPIA, http://gepia.cancer-pku.cn/) database with basis of TCGA database. The results were similar to these findings (Figure 1C). Kaplan-Meier survival curve results showed expression levels of ZC3HAV1 were negatively related to total patients’ survival with PC (Figure 1D). We next performed qRT-PCR and Western blot to check the expression level of ZC3HAV1 mRNA as well as protein in pancreatic cell lines PANC-1, CFPAC, SW1990, MIA PaCa-2 and the pancreatic duct epithelial cell line (HPDE). The expression levels of ZC3HAV1 mRNA and protein were up-regulated in PC cell lines compared with HPDE cells (Figure 1E, 1F). Then, we analyzed the mutual relation between ZC3HAV1 expression and clinical features of the PC patients (Table 1). Results showed that the expression of ZC3HAV1 is significantly positive correlation of larger tumor diameter (P= 0.028) together with lymph node metastasis (P = 0.03). Such results showed ZC3HAV1 might have an impact on PC cell growth and metastasis. The PANC-1 and MIA PaCa-2 cell lines exhibited the highest levels of ZC3HAV1 expression and were selected as the candidate for subsequent experiments.

**Table 1:** General clinicopathological characteristics of patients.

**Characteristic**		**ZC3HAV1**		***p value***
		**Low 14**	**High 16**	
Age	≤50	8	9	0.961
	>50	6	7	
Gender	male	7	10	0.491
	female	7	6	
Diameter	≤2	10	5	0.028*
	>2	4	11	
Grade	I/II	5	8	0.431
	III/IV	9	8	
Lymphatic	Negative	9	4	0.03*
	Positive	5	12	
Distant	Negative	10	12	0.825
	Positive	4	4	
TNM stage	I/II	9	13	0.295
	III/IV	5	3	

### ZC3HAV1 promotes PC cell proliferation and G1/S phase transition *in vitro*

**Figure 2:**
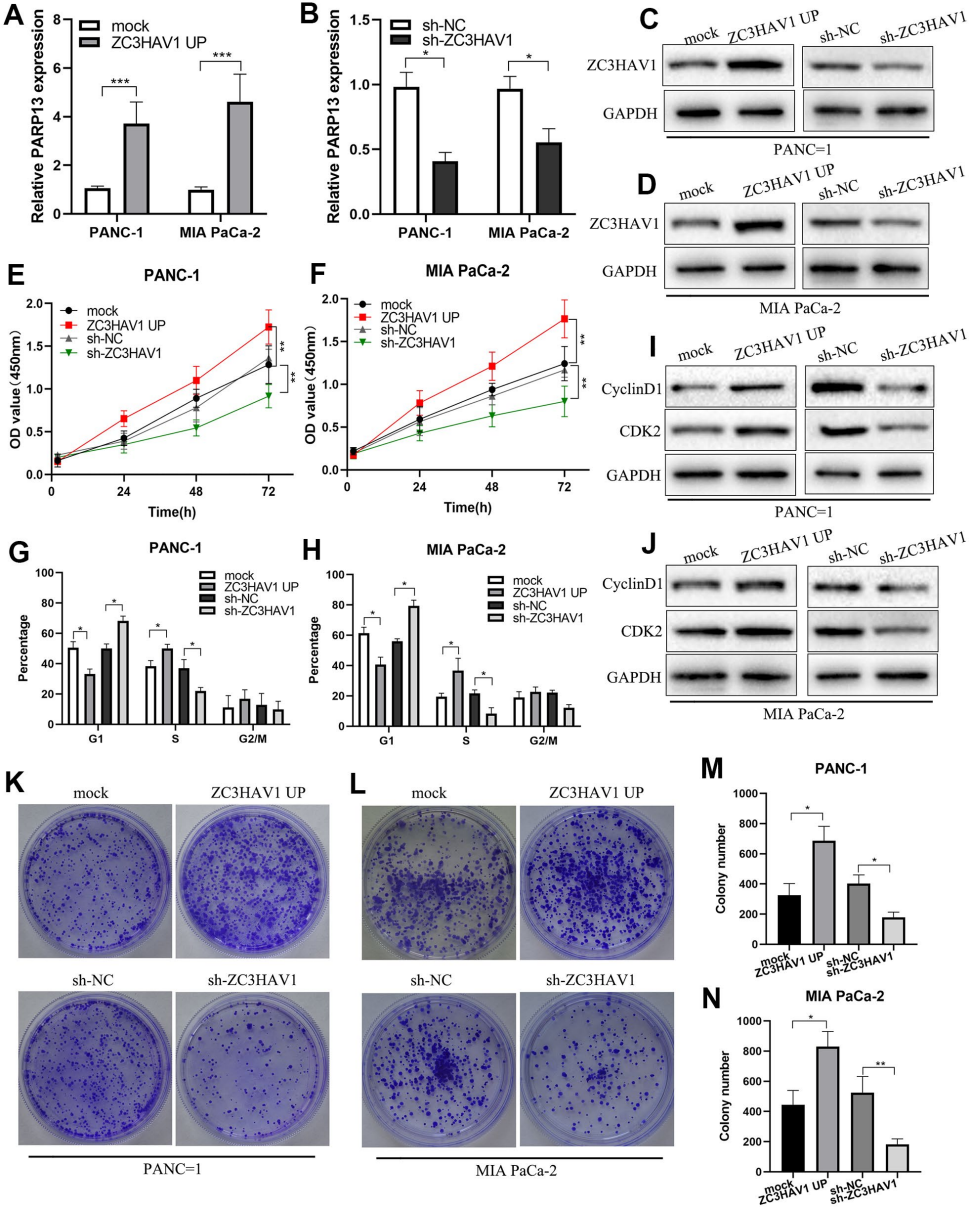
**ZC3HAV1 improves pancreatic cancer cell proliferation and G1/S phase transition.** (**A**–**D**) The transfection efficiency of ZC3HAV1 was measured by qRT-PCR and western blot in pancreatic cancer cells. (**E**, **F**) The effect of ZC3HAV1 on the proliferation of pancreatic cancer cells was found by CCK-8 assay. (**G**, **H**) Flow cytometry dissection of the cell cycle in pancreatic cancer cells after transfection was done. (**K**–**N**) Carried out cell colony formation assay in PANC-1 and MIA PaCa-2 cells after transfection. (**I**, **J**) Western blotting shows cell cycle protein Cyclin D1 and CDK2 expression in PANC-1 and MIA PaCa-2 cells after transfection. Data appeared to be average ± SD, *P< 0.05, **P< 0.01.

To more closely examine the function of ZC3HAV1 in PC, we used lentivirus vectors for constructing stable ZC3HAV1 overexpression or ZC3HAV1 downregulation PANC-1 and MIA PaCa-2 cell lines. The transfection effect of ZC3HAV1 in both cell lines was confirmed by western blot and qRT-PCR analysis (Figure 2A–2D). CCK8 assays and colony forming assays revealed the upregulation of ZC3HAV1 exerted a facilitative effect on the growth and clonogenicity of PANC-1 and MIA PaCa-2 cells compared with negative control (mock and sh-NC) groups, while downregulation of ZC3HAV1 lead to decreasing growth and reducing clonogenicity of both cell lines (Figure 2E, 2F, 2K–2N). Because enhanced cell proliferation is often caused by deregulation of the cell cycle, we checked if cell cycle profiles were altered after changed expression of ZC3HAV1. According to flow cytometry analysis, overexpression of ZC3HAV1 dramatically increased the percentage of cells in S stage in comparison to controls, while the percentage of cells in G1 stage reduced. In contrast, inhibition of ZC3HAV1 greatly reduced the percentage of cells in S stage, while the percentage of cells in G1 stage improved (Figure 2G, 2H). We also evaluated the roles of ZC3HAV1 played in the expression of various cell cycle-related proteins by western blotting. Compared with controls, ZC3HAV1 overexpression up-regulated the protein expression of Cyclin D1 along with CDK2, while ZC3HAV1 knockdown took an adverse impact (Figure 2I, 2J).

### ZC3HAV1 increases migration and invasion of PC cells

**Figure 3:**
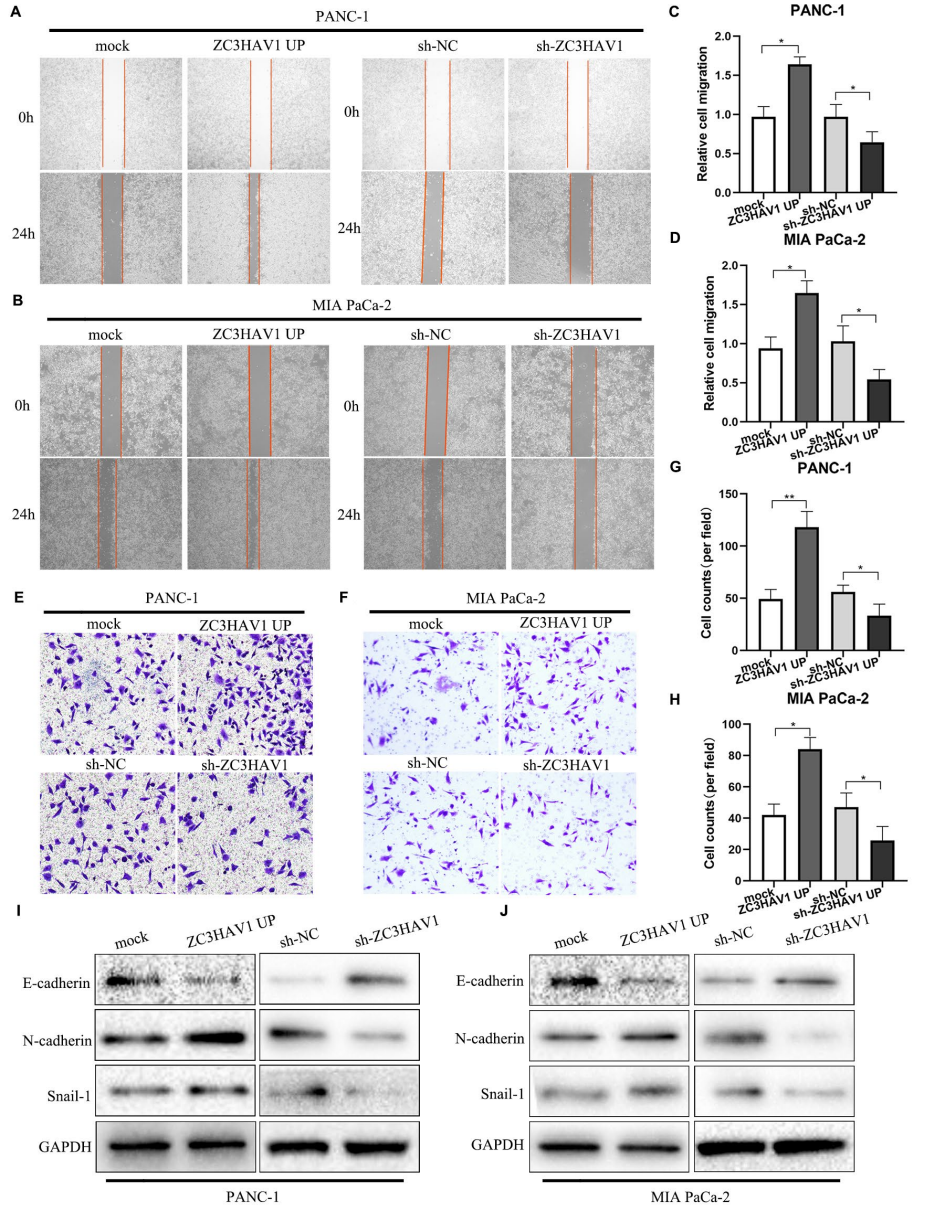
**ZC3HAV1 increases the migratory ability in PANC-1 and MIA PaCa-2 cells.** (**A**–**D**) Migration of cells after transfection was measured by wound-healing assays. (**E**–**H**) Cell invasion abilities were settled by transwell assays after transfection. (**I**, **J**) Western blot analysis of the EMT-related markers E-cadherin, N-cadherin, and Snail-1 after transfection in PANC-1 and MIA PaCa-2 cells. Data were showed by mean ± SD, *P< 0.05, **P< 0.01.

Migration and invasion exert a vital influence on PC development. Therefore, the roles of ZC3HAV1 played in migration and invasion of PC cells were evaluated by carrying out wound healing and transwell experiments. In addition, the outcome reflected the migration along with invasion abilities of PANC-1 along with MIA PaCa-2 cells significantly increased due to upregulation of ZC3HAV1 while markedly inhibited by down-regulation of ZC3HAV1 (Figure 3A–3H). Activation of the epithelial-mesenchymal transition (EMT) plays a key part in PC cell metastasis. Subsequently, we examined the expression of EMT-relating proteins like E-cadherin, N-cadherin and Snail-1 by Western blotting. According to Figure 3I, 3J, ZC3HAV1 knockdown raised expression of the E-cadherin and decreased expression of N-cadherin and Snail-1, while ZC3HAV1 overexpression took the adverse impact. In addition, the mRNA expressions of these EMT markers were measured using qRT-PCR analysis, the results also supported the protein expression results (Supplementary Figure 1).

### Knockdown of ZC3HAV1 attenuated the tumorigenic ability of PC *in vivo*

**Figure 4:**
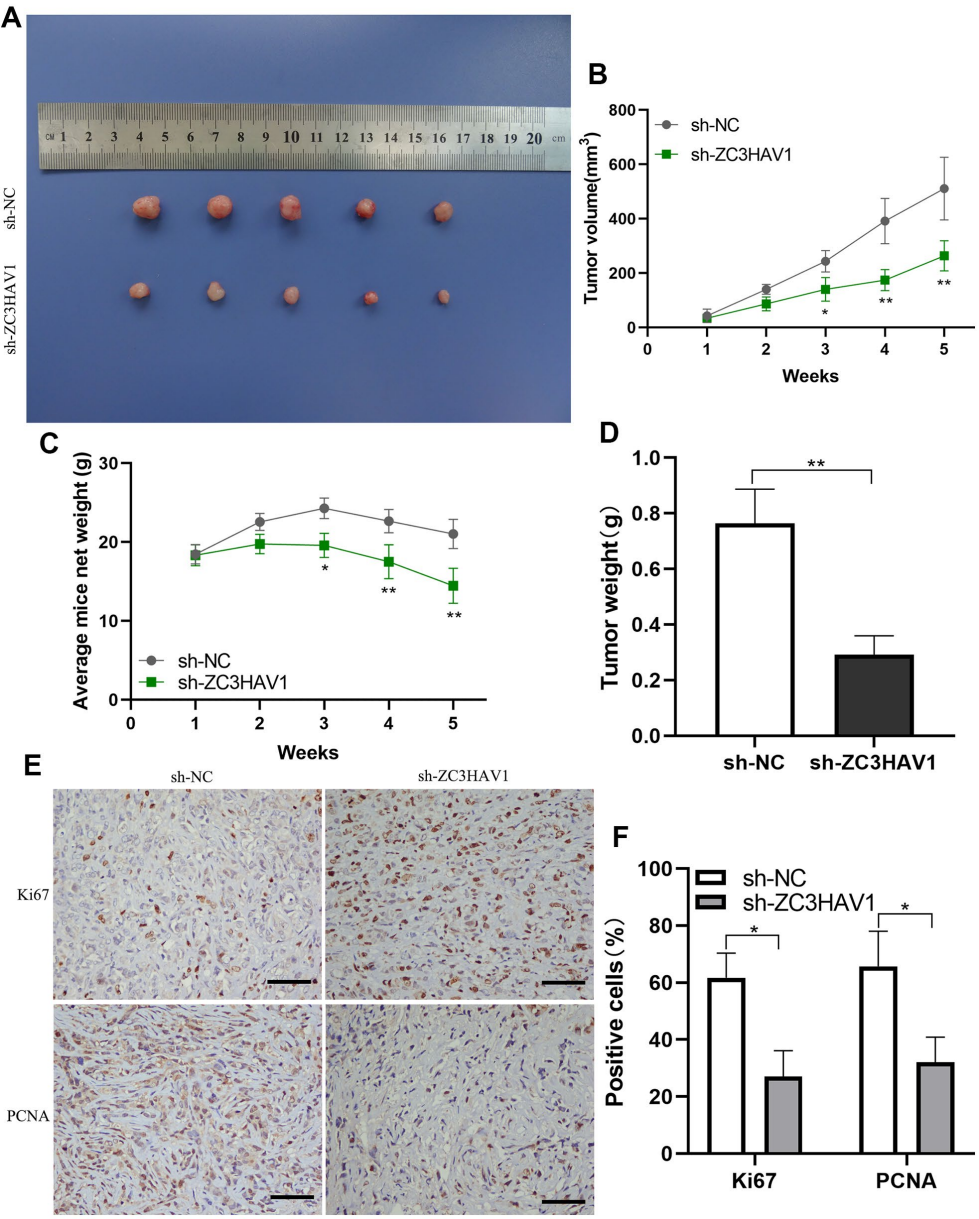
**ZC3HAV1 knockdown decreases the proliferation of pancreatic cancer *in vivo*.** (**A**) In the end of the experiment, all tumors were resected and the tumors in the ZC3HAV1-silencing groups apparently appeared to be smaller than those in the control group. (**B**) The volumes of the tumor were measured every week. (**C**) The weight of mice in the ZC3HAV1 knockdown and sh-NC group was detected every week. (**D**) In the end of experiment, the weight of the xenografts were measured. (**E**, **F**) The expression of Ki67 along with PCNA in xenografts was detected through immunohistochemical staining (scar bar: 100 μm). Data speared to be mean ± SD, *P< 0.05, **P< 0.01.

To more closely find out the function of ZC3HAV1 in tumor growth *in vivo*, the PANC-1 cells with stable down-expression of ZC3HAV1 or control cells were injected into nude mice subcutaneously. After that, the tumor volume together with weight of mice was monitored weekly. Compared with control mice, tumors derived from ZC3HAV1-knockdown cells developed obviously smaller and lower (Figure 4A, 4B). Moreover, the weight of mice rapidly reduced in the ZC3HAV1-knockdown group and the weight of sh-ZC3HAV1-derived tumors showed a marked decrease in comparison to the control group (Figure 4C, 4D). Furthermore, histological analysis revealed that Ki-67 and PCNA staining decreased in the tumors from ZC3HAV1-knockdown group in comparison to control tumors (Figure 4E, 4F).

### ZC3HAV1 regulates the ERK pathway by targeting KRAS

**Figure 5:**
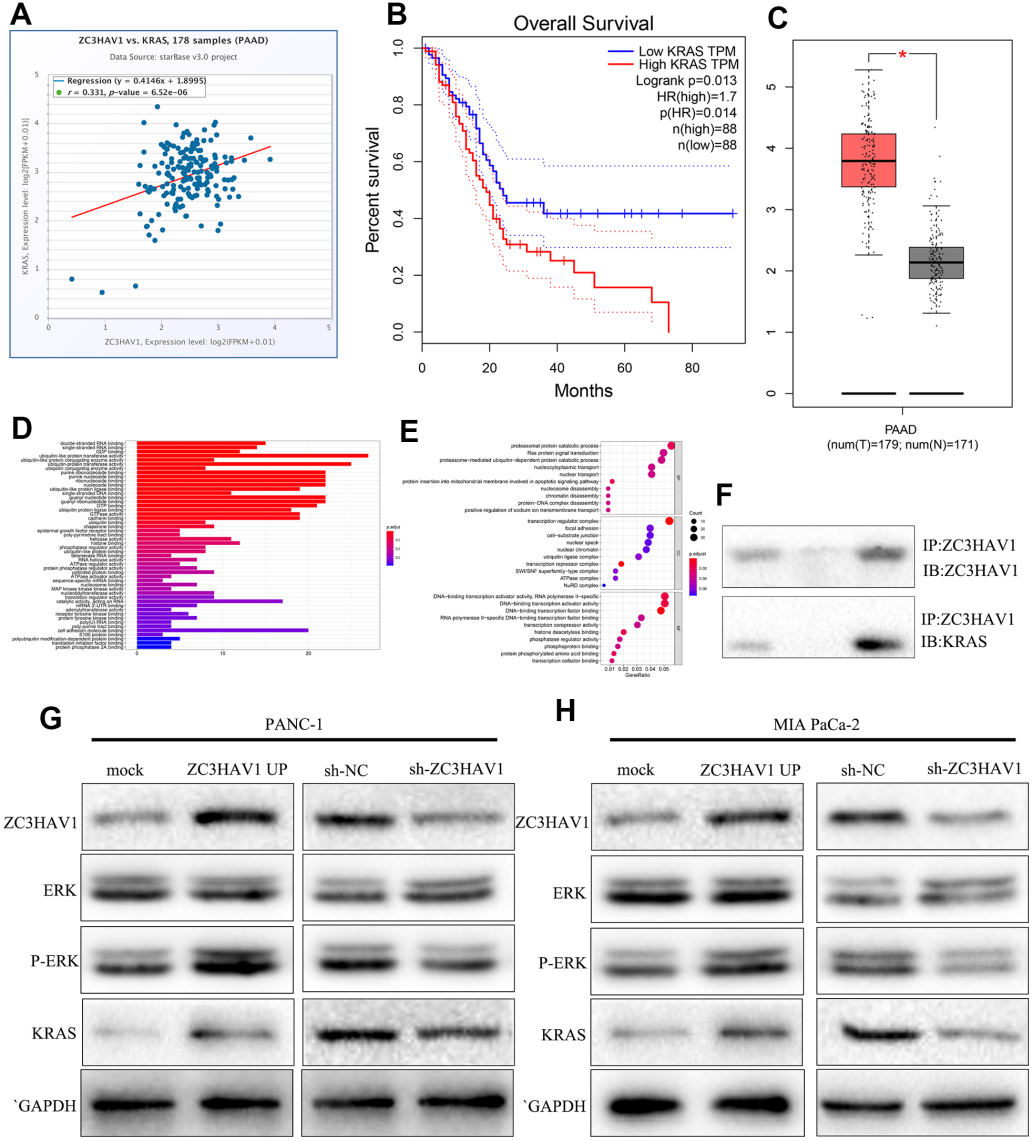
**ZC3HAV1 regulates the ERK pathway by targeting KRAS.** (**A**, **B**) KRAS correlation with ZC3HAV1 and its associated with overall survival were predicted by starBase. (**C**) The expression of KRAS in PC tissues compared with adjacent normal tissues with basis of the GEPIA database. (**D**, **E**) GO analysis of KRAS was performed. (**F**) ZC3HAV1 protein was immunoprecipitated with ZC3HAV1 antibody and levels of bound KRAS were found by western blotting. (**G**, **H**) Protein expression levels of vital modulators of the ERK signaling pathway and dissected KRAS by western blotting.

As we all known, KRAS exerts a vital regulatory influence on the occurrence of tumors. We predicted a positive correlation between ZC3HAV1 and KRAS through starBase (Figure 5A). And we also predicted that KRAS was up-regulated in PC and relation to poor prognosis (Figure 5B, 5C). The immunoprecipitation assay was performed to test our prediction. The result showed that ZC3HAV1 coimmunoprecipitated with ERK (Figure 5F). We predicted that ZC3HAV1 could regulate ERK pathway through GO analysis (Figure 5D, 5E). After that, we tested our prediction by Western blot. The result indicated that ZC3HAV1 overexpression increased p-ERK and KRAS expression, while ZC3HAV1 knockdown inhibited p-ERK and KRAS expression in PC cells (Figure 5G, 5H).

### ZC3HAV1 regulates proliferation and migration of PC cells via KRAS

**Figure 6:**
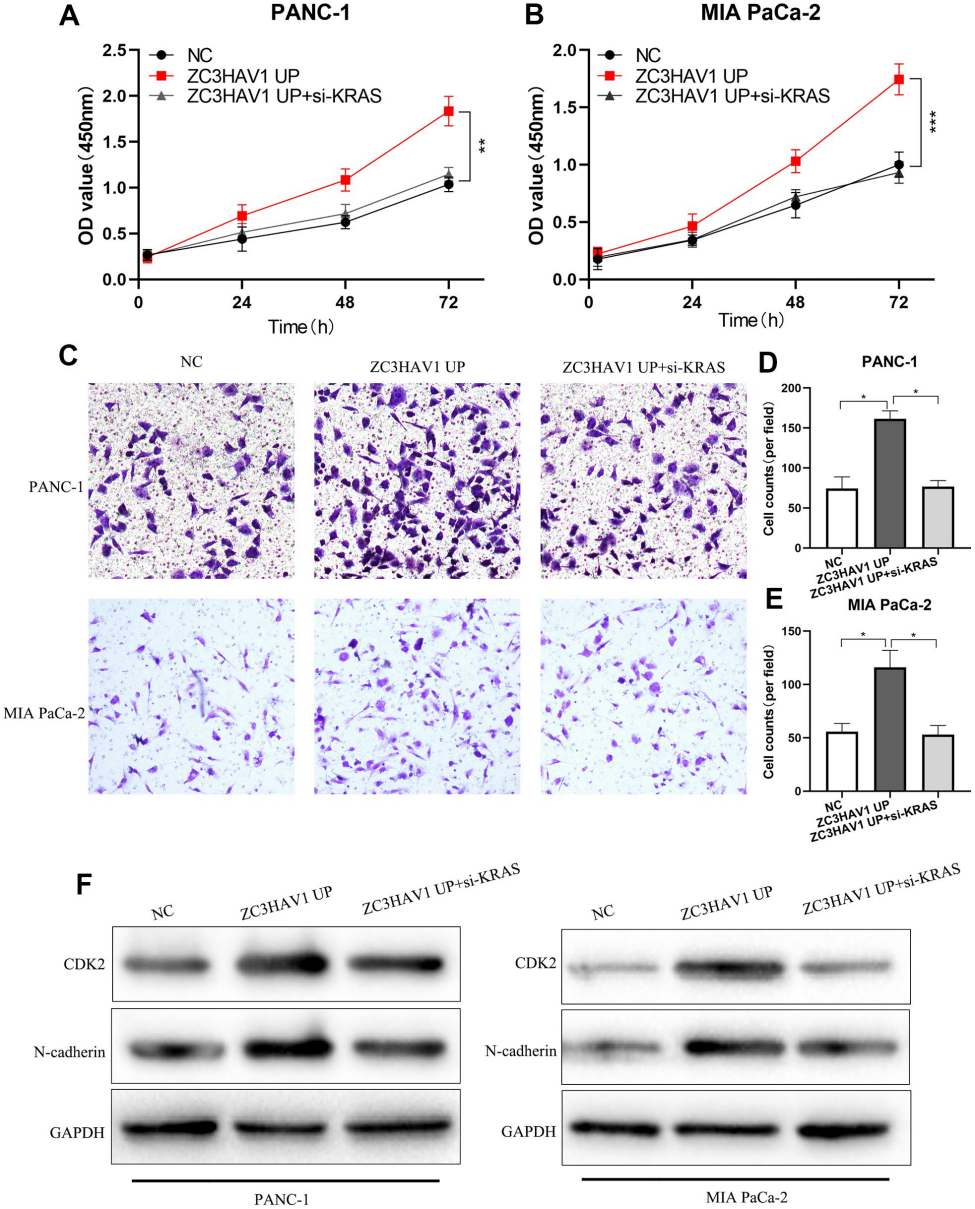
**After down-regulating KRAS, the promotion roles of ZC3HAV1 played in the proliferation and invasion of PC cells was weakened.** (**A**, **B**) CCK8 assays were employed to find out the proliferation ability of every group. (**C**–**E**) Performed transwell assay in order to examine the invasion ability of each group. (**F**) The related expression of proteins of proliferation (CDK2) and EMT (N-cadherin) was analyzed by Western blotting. Data appeared to be mean ± SD, *P< 0.05, **P< 0.01.

To demonstrate whether KRAS was engaged with the part of ZC3HAV1 played in proliferation and metastasis, CCK-8 and transwell experiments were conducted to check the functional impacts of knockdown of KRAS integrated with or without ZC3HAV1 UP. ZC3HAV1 overexpression markedly increase the cell proliferative capacity, whereas KRAS knockdown could reverse the phenomenon (Figure 6A, 6B). Similar to these findings, transwell experiment showed knockdown of KRAS attenuated ZC3HAV1 promoted cell invasion (Figure 6C–6E). After that, ZC3HAV1 overexpression remarkably increased cell cycle-associated proteins CDK2 and N-cadherin levels, while KRAS knockdown could alter the increase of these proteins via ZC3HAV1 (Figure 6F).

## DISCUSSION

PC is a devastating disease and its survival rate in five years is less than 5%. The incidence rate is rising annually. In America, PC is becoming the second most universal reason of cancer-associated death in 2030 [17]. Thus, better knowledge of the molecular mechanisms related to the PC progression is critical to identify new treatment strategies. With the development of gene detection technology, more and more oncogenes and anti-oncogenes have been found to be involved in the occurrence of PC. For example, it was reported that silenced ZNF154 was in relation to longer survival in resectable PC [18]. Besides, SPOP suppressed PC progression by promoting the degradation of NANOG [19]. Moreover, TRAF6 was over-expressed in PC and improved the tumorigenicity of PC cells [20]. ZC3HAV1 belongs to the PARP family of proteins. Several reports have indicated that the PARP family members are functional differently in multiple cancers. For instance, PARP6 is expressed at low levels in colorectal cancer. PARP14 is upregulated in hepatocellular carcinoma [21, 22]. Moreover, ZC3HAV1 has been found to engaged with the occurrence of multiple cancers, like liver cancer, colon cancer and bladder cancer [6]. However, few studies have evaluated the function of ZC3HAV1 in cancer, and no study has focused on the clinical meaning and effect of ZC3HAV1 in PC.

Our studies showed the level of ZC3HAV1 was greatly upregulated in PC tissues and cell lines. Meanwhile, overexpression of ZC3HAV1 had positive relation with larger tumor size and lymph node metastasis together with overall poor survival of patients with PC. More *in vivo* and *in vitro* functional experiments showed ZC3HAV1 greatly improved the proliferation along with metastasis of PC cells, and down-regulation of ZC3HAV1 exhibited an adverse impact. Besides, our result showed that ZC3HAV1 coimmunoprecipitated with KRAS. It has been known that KRAS takes a vital regulatory part in the proliferation, invasion and metastasis of PC. The findings reveal that ZC3HAV1 exerts an oncogene impact on the development of PC. It might be a new diagnostic and prognostic marker or therapeutic target in patients with PC.

The MAPK pathway is a signal transduction pathway that has been studied in depth, and it exists widely in various cells of the human body. And it is strongly linked to the proliferation, apoptosis, invasion and migration of tumor cells [23]. Previous studies have found that KRAS is activated in most patients with PC, and this persistent abnormal KRAS can activate the MAPK signaling pathway [24]. ERK is the core molecule in the MAPK pathway, and it forms an efficient and accurate signal transduction system with upstream activating molecules and downstream effectors. It is activated by phosphorylation to produce activated P-ERK, which in turn activates the entire pathway, thereby regulating many downstream protein kinases, phospholipases and transcription factors, and exerting important biological functions [25]. Abnormal cell proliferation serves as one of the most important features of tumors. Cyclins and their regulatory proteins (CDKs) can regulate the cell cycle, thereby affecting cell proliferation. The most important molecules are Cyclin-D1 and CDK2, which are essential proteins for tumor cells to enter the G1 phase to the S phase and to switch from the S phase to the G2 phase. Cyclin D1 can combine with CDK to form a complex, promote the transition of cells from G1 phase to S phase, and accelerate the cell cycle process. The activity of CDK2 can regulate the rate of the cell cycle. Highly active CDK2 can promote cells to quickly enter the division phase, while low-active CDK2 can make cells enter a resting state similar to G0 phase [26, 27]. Our results found that after interfering with the expression of ZC3HAV1, it could affect the expression of cell cycle-associated proteins cyclin D1 and CDK2, thereby affecting the transition of cells from G1 to S phase, and it finally effectively regulated the cell cycling process. Epithelial-mesenchymal transition (EMT) is a procedure where epithelial cells lose their cell polarity and adhesion to obtain the features of invasion and metastasis [28, 29]. The studies revealed that high expression of ZC3HAV1 significantly promoted the protein expression levels of E-cadherin, N-cadherin and Snail-1, which were key markers in EMT and strongly linked to the invasion and metastasis of PC. Subsequently, we found that ZC3HAV1 could directly bind to KRAS and upregulate the expression of KRAS. Additionally, overexpression of KRAS activates ERK signaling to promote PC proliferation and metastasis. Therefore, we speculated that ZC3HAV1 directly targeted KRAS, thereby affecting the MAPK pathway to perform the above-mentioned effects.

Generally speaking, we first proved that ZC3HAV1 was upregulated in mankind PC cell lines and clinical tissue samples. Furthermore, we discovered that ZC3HAV1 regulated the MAPK Pathway by aiming at KRAS to promote the PC cell lines proliferation and invasion. Our findings revealed that ZC3HAV1/KRAS/ERK signaling axis might be a biomarker or target for PC diagnosis and treatment.

## MATERIALS AND METHODS

### Tissues

30 pairs of PC tissues and their corresponding adjacent tissues were gathered by us from patients receiving an operative treatment in Affiliated Hospital of Guizhou Medical University (Guiyang, China). Then we stored these samples in liquid nitrogen with the temperature of -80° C. All patients were clear about the situation and reached a consensus. The tissue specimens were given evaluation and identification by an pathologist with experience, and tumorous cellularity was examined by cryostat sectioning together with dissection of most cell area. We recorded the clinical data including gender, age, tumor size, clinical stage as well as lymph node metastasis. This research got approval from the Human Research Ethics Committees at Affiliated Hospital of Guizhou Medical University.

### The starBase and GEPIA database

The expression of ZC3HAV1 and KRAS in PC was analyzed with the basis of the Gene Expression Profiling Interactive Analysis (GEPIA) (http://gepia.cancer-pku.cn/). The prognostic values of KRAS regarding differential expression and survival analysis were analyzed by starbase (http://starbase.sysu.edu.cn/). The correlation analysis of ZC3HAV1 and KRAS were also analyzed by starBase.

### Cell lines and cultures

The PC cell lines including CFPAC, SW1990, PANC-1 and MIA-PaCa-2 along with pancreatic ductal epithelium cell line HPDE were bought and they have been identified by STR typing from ATCC. PANC-1 and MIA-PaCa-2 grew in DMEM medium (Gibco, NY, USA) added with 10% fetal bovine serum (Gibco), 100 μg/ml streptomycin and 100 U/ml penicillin G (Sigma, MO, USA). CFPAC, SW1990 and HPDE cells grew in 1640 medium (Gibco) with supplements of 100 μg/ml streptomycin, 100 U/ml penicillin G and 10% fetal bovine serum with temperature of 37° C. Every cell line was contained with temperature of 37° C in a humidified incubator full of 5% CO_2_.

### Immunohistochemistry

The collected tissue of human beings and mice were fixed, embedded and cut into 5μm slices, which were roasted in an oven at 80° C for half an hour. Dewaxing with xylene and different concentrations of alcohol (100%, 95%, and 85% for five minutes each). Then stained the sections with hematoxylin and eosin. After that, the sections were deal with citric buffer and incubated in phosphate-buffered saline (PBS). Then, primary antibodies (Ki67, PCNA and ZC3HAV1) and secondary antibody underwent all-night incubation at 4° C. In addition, the sections were visualized and counterstained with hematoxylin. At last, observe and take pictures under a microscope.

### Quantitative real-time reverse transcription-PCR (qRT-PCR)

In line with guidelines of manufacturer, extracted total RNA from cells and tissues via TRIzol Reagent (Invitrogen). The concentrations of total RNA were measured by use of a Nano Drop ND-1000 spectrophotometer (Nano Drop Technologies) at 260 and 280 nm (A260/280), and through an Agilent 2100 Bioanalyzer (Agilent Technologies) the concentrations were checked. In line with the protocol of manufacturer, quantitative real-time reverse transcription PCR (qRT-PCR) assays were carried out employing a TaqMan miRNA Assay (Applied Biosystems).

### Generation of stable cell lines and antibodies

Human ZC3HAV1 over-expressing (ZC3HAV1 UP), ZC3HAV1 knock-down (sh-ZC3HAV1) and the control (mock and sh-NC) lentivirus were purchased from Genechem (Shanghai, China). The sequences of the forward primer and reverse primer of ZC3HAV1 was as follows:5'-CCGGTGCAACTATTCGCAGT-3' and 5'-TCAGTCCAGAGAGTTCGTGATTT-3'. All of transfections were performed in accordance with instructions of manufacturers. The antibodies (ZC3HAV1, CyclinD1, CKD2, N-cadherin, E-cadherin, Snail-1, ERK, P-ERK and KRAS) were used in our study purchased from CST and Sigma.

### Cell proliferation assay

The test was put on the cell viability by the Cell Counting Kit-8 (Dojindo, Tokyo, Japan). At 48 h after transfection was done, cells were re-seeded in 96-well plates at 2 × 10^3^ cells/well. Every analysis was performed in five wells; cells were incubated for at 1 d, 2 d, and 3 d. Next, each well was added with CCK-8 (10 μl) and cells underwent one-hour incubation. A microplate reader was applied to obtain absorbance at 450 nm. The average value was settled after repeating the experiment for three times.

### Colony formation assay

Transfected cells were seeded in 6 cm dish at approximately 1.5 × 10^3^/well and cultured in DMEM medium with 10% FBS with temperature of 37° C for about 14 days. Cells were maintained for 20 minutes with 4% paraformaldehyde and stained for 30 minutes with 0.1% crystal violet. Colonies were counted under the microscope from seven random fields. The experiments were performed repeatedly at least for three times.

### Flow cytometry

Cells were synchronized by serum starvation for 48 h and then replated in 10% FBS medium for 3 h. Then cells were collected and fixed in 75% ethanol at 4° C all night, and before staining they were washed twice with PBS. On the grounds of the protocol of manufacturer, the PI/RNase Staining Buffer (BD Biosciences, USA) was applied to stain DNA. Stained cells were conducted with analysis via a flow cytometer. All assays were performed repeatedly at least three times.

### Wound-healing assay

Plant the processed cells into a six-well plate, observe the cells under the microscope so that the desired density is reached, and draw a cross mark with the tip of a 200μl pipette against the ruler. Use PBS to wash the scratched cell debris and take pictures. Change to serum-free medium and continue culturing for 24 hours. Take out the six-well plate, wash it with PBS and take a picture to observe the healing of the scratches.

### Transwell assay

The treated cells were planted into the upper chamber of serum-free medium. The upper chamber was covered by Matrigel, and the lower chamber medium had 10% fetal bovine serum. After culturing for 24 hours, the cells of the upper chamber were removed. The cells passing through the substrate were fixed by 4% paraformaldehyde, then stained with crystal violet at indoor temperature, and counted by a microscope.

### Animal experiments

We purchased female athymic nude mice (4 weeks old) from the Shanghai Experimental Animal Center of Chinese Academy of Sciences (Shanghai, China). Female athymic NCr-nu/nu nude mice were inoculated with PANC-1 cells with transfection of knock-down (sh-ZC3HAV1) or negative control (NC) lentivirus. Mice were then euthanized and sizes of tumor were calculated at the sixth week after injection. Every tumor was collected, weighed, and imbedded into paraffin for immunohistochemical (IHC) staining. All of the animal tests were conducted in line with the protocol with approval of the Animal Committees of the Affiliated Hospital of Guizhou Medical University.

### Western blot analysis

PBS was employed to wash cells and in RIPA buffer cells got lysed. Protein concentration was settled by the BCA protein test, then 10% sodium dodecyl sulfate-polyacrylamide gel electrophoresis (SDS-PAGE) were employed to split equivalent proteins, and the equivalent proteins were moved to polyvinylidene fluoride (PVDF) membranes (Millipore, Billerica, MA, USA). Membranes were covered with 5% fat-free milk buffer and incubated all night at temperature of 4° C with primary antibodies, and then with secondary antibody for one hour at indoor temperature. Via Enhanced Chemiluminescence (ECL) bands were visualized and by use of a gel imaging system the analysis of the bands were conducted. GAPDH was applied as loading control.

### Immunoprecipitation (IP)

The processed cells are collected adding into IP lysis buffer (having protease inhibitor) and are lysed on ice for half an hour. Then take the supernatant after centrifugation. Both 1 μg corresponding antibody and 10-50 μl protein A/G-beads are added into the cell lysate and incubate with slow shaking at 4° C for all night. Then centrifuge the protein A/G-beads to the bottom of the tube after immunoprecipitation, remove the cleansing with caution, and wash the protein A/G-beads by 1ml lysis buffer for three to four times. In the end, the loading buffer is added to boil for ten minutes. Western blotting is used for mass spectrometry analysis.

### Statistical analyses

Data appear to be average ± SD. After at least three independent experiments the data were gained. Paired Student’s t-test was designed for difference comparing between the two groups. Differences shown in experiments of proliferation were analyzed by one-way ANOVA. A P value of <0.05 was taken meaningful on the aspect of statistics. Every analysis was conducted by use of SPSS 22.0 statistical software (SPSS, Chicago, IL, USA).

## Supplementary Material

Supplementary Figure 1
