# Supplementary material for: ZC3HAV1 promotes the proliferation and metastasis via regulating KRAS in pancreatic cancer
Source: Aging (Albany NY). 2021 Jul 28;13(14):18482–97. doi: 10.18632/aging.203296 (PMC8351712; doi:10.18632/aging.203296)
Supplement: Supplementary Figure 1 [file aging-13-203296-s001.pdf]

SUPPLEMENTARY FIGURE

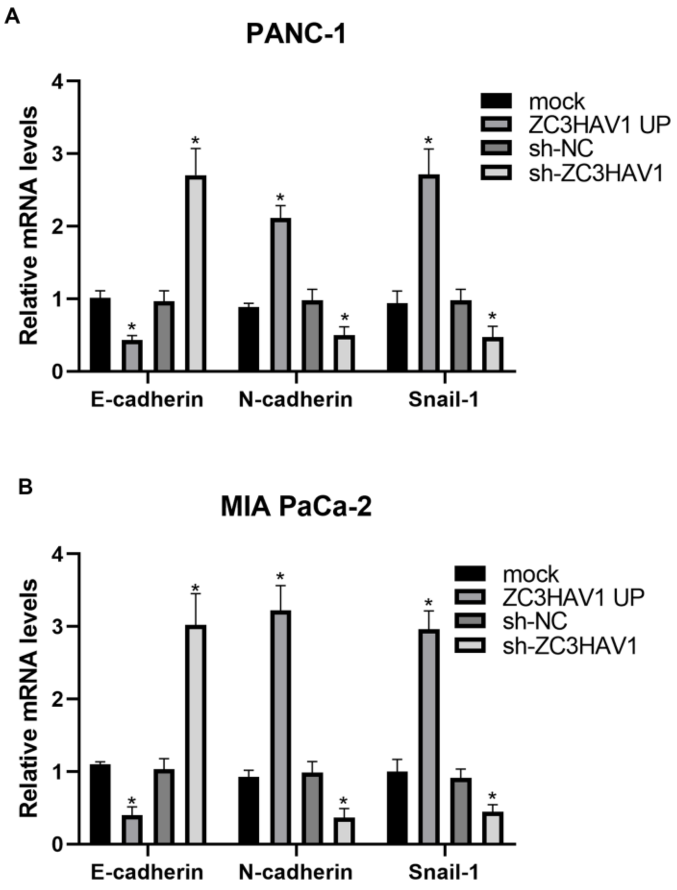

Supplementary Figure 1. ZC3HAV1 promotes the mRNA expression of EMT-associated markers E-cadherin, N-cadherin, and Snail-1. (**A**) PANC-1 cell lines. (**B**) MIA PaCa-2 cell lines.
